# Experiences of Autism Acceptance and Mental Health in Autistic Adults

**DOI:** 10.1007/s10803-017-3342-7

**Published:** 2017-10-25

**Authors:** Eilidh Cage, Jessica Di Monaco, Victoria Newell

**Affiliations:** Department of Psychology, Royal Holloway, University of London, Egham Hill, Egham, Surrey TW20 0EX UK

**Keywords:** Autism acceptance, Mental health, Masking, Camouflaging

## Abstract

Mental health difficulties are highly prevalent in individuals on the autism spectrum. The current study examined how experiences and perceptions of autism acceptance could impact on the mental health of autistic adults. 111 adults on the autism spectrum completed an online survey examining their experiences of autism acceptance, along with symptoms of depression, anxiety and stress. Regression analyses showed that autism acceptance from external sources and personal acceptance significantly predicted depression. Acceptance from others also significantly predicted stress but acceptance did not predict anxiety. Further analyses suggested that experiences of “camouflaging” could relate to higher rates of depression. The current study highlights the importance of considering how autism acceptance could contribute to mental health in autism.

## Introduction

Autism is a lifelong neurodevelopmental condition which affects the way individuals process the world; autistic^1^ individuals show differences in their social communication, social interactions, sensory sensitivities, along with restricted and repetitive interests and behaviours (APA 2013). The prevalence of comorbid mental health conditions in autism is strikingly high. For example, Eaves and Ho (2008) found that 77% of young autistic adults in their sample had additional mental health diagnoses, including anxiety, depression and bipolar disorder. Other studies suggest that the prevalence of depression in autistic individuals is around 34% (Stewart et al. 2006). Anxiety is also frequently found to be higher in the autistic population than within the non-autistic population (Gillott and Standen 2007), alongside a higher prevalence of social anxiety disorder (Maddox and White 2015). Difficulties with mental health are consequently thought to contribute to a poorer quality of life in autism (Robertson 2009).

Given this high prevalence of mental health difficulties, it is vital to understand why those on the autism spectrum are at a higher risk. There are a number of possible factors that could contribute to this prevalence. The current study adopts the “social model approach” as a means of explaining mental health comorbidity in autism. The social model claims that factors external to the individual cause disabling features (Shakespeare 2006)—for example, an employer’s attitudes or lack of understanding of autism could prevent autistic individuals from finding employment, rather than difficulties an individual may experience as a result of autism itself. When applying the social model to mental health in autism, one factor—and the focus of the current study—that could impact on mental health is an individual’s experiences and perceptions of autism acceptance. Other factors which may affect mental health in autism may include sensory sensitivities (Green and Ben-Sasson 2010) and intolerance of uncertainty (Maisel et al. 2016). The current study focused on autism acceptance as it has been little explored as a risk factor for mental health issues in autistic adults.

Autism acceptance can be defined as an individual feeling accepted or appreciated as an autistic person, with autism positively recognised and accepted by others and the self as an integral part of that individual. Autism acceptance from others could be important for autistic individuals’ mental health for a number of reasons. Within the mental health literature, perceived stigma from others is thought to contribute negatively to the mental health of stigmatised groups (Mak et al. 2007). In terms of stigma against autistic people, Sasson et al. (2017) found that non-autistic individuals tend to make rapid unfavourable judgements about those on the autism spectrum. In their study, neurotypical participants rated pictures or videos without knowing that some of the people in the videos were on the autism spectrum. Findings showed that the autistic people were rated as less likeable, less attractive and that the participant would be less likely to engage with them socially, suggesting that societal acceptance may be poor. Moreover, when interviewed about their experiences of acceptance from society, adults on the autism spectrum reported that a lack of public understanding was contributing to their experiences of social isolation and anxiety (Griffith et al. 2012). Studies examining autism acceptance additionally indicate that although non-autistic people are aware of autism (Dillenburger et al. 2013, 2015), misunderstandings and misconceptions are common nonetheless, such as believing that changing an autistic child’s diet can lessen symptoms, or that autism can be outgrown (Tipton and Blacher 2014). Further, unconscious biases towards autism can still be prevailingly negative even in those who work regularly with autistic children (Kelly and Barnes-Holmes 2013).

In terms of acceptance from closer social networks, having the opportunity to engage with others who are like-minded and to develop one’s sense of belonging is thought to be important to the well-being of autistic adults (Milton and Sims 2016). Indeed, sense of belonging is argued to be vital to the well being of all individuals, irrespective of autism (Baumeister and Leary 1995). Research has also suggested that previous experience of contact with autistic individuals is associated with greater autism acceptance in university students (Gardiner and Iarocci 2014) and those with a family member on the autism spectrum tend to be more accepting and open towards autism (Nevill and White 2011). Longitudinal studies have also shown that autistic individuals believe supportive family and friends help them to develop greater feelings of self-worth (Hurlbutt and Chalmers 2002). Equally, parents of autistic children who demonstrate higher acceptance of their child’s autism have been found to have fewer mental health problems (Weiss et al. 2012). Additionally, in a study where loneliness was positively correlated with anxiety and depression, individuals on the autism spectrum who reported having more friends experienced fewer feelings of loneliness, as well as fewer anxious and depressive symptoms (Mazurek 2014). As such, it may be that feeling accepted by others could act as a protective factor against mental health problems.

Personal acceptance is also an important variable within mental health—for example, unconditional self-acceptance in non-clinical samples has been shown to negatively correlate with anxiety (Chamberlain and Haaga 2001) and depression (Flett et al. 2003). Many autistic people want to be accepted for being “who they are” and take pride in being neurodivergent, a term (alongside neurodiversity) used to describe differences in the way people think, with diversity in the brain an important and celebrated part of human variation (Cage et al. 2016a; Humphrey and Lewis 2008; Hurlbutt and Chalmers 2002; Jaarsma and Welin 2012; Robertson 2009). Therefore, the current study also considered personal acceptance of being on the autism spectrum. Kapp et al. (2013) noted that those who self-identified more strongly with the concept of neurodiversity tended to view autism itself more positively. Recent research has also indicated that identifying positively with an autistic identity mediates the relationship between self-esteem and mental health difficulties, suggesting that personal acceptance of autism as part of one’s identity could protect against depression and anxiety (Cooper et al. 2017). Despite this, many autistic individuals frequently report “masking” or “camouflaging”—that is, they may use strategies to camouflage the fact they are on the autism spectrum in order to “fit in” to the non-autistic world (Dean et al. 2016; Hull et al. 2017). Camouflaging one’s identity as an autistic person could have a subsequent impact on experiences of acceptance, if one is not “out” as being on the autism spectrum.

To the best of our knowledge, no research has directly examined autistic adults’ perceptions and experiences of autism acceptance and their relation to mental health difficulties. It is not known how autism acceptance from both self and others relates to mental health outcomes for autistic adults. The current research thus aimed to test the relationship between perceived autism acceptance and mental health (specifically, depression, anxiety and stress) in a sample of autistic adults. We hypothesised those autistic adults who experienced less autism acceptance would show greater prevalence of the symptoms of depression, anxiety and stress.

## Methods

### Participants

Autistic adults over the age of 18 were recruited via a range of means; these included sharing a link to the online survey on social media or through autism organisations and groups in the UK. A link to the survey was provided for individuals wishing to participate. Groups and organisations were initially contacted with an explanation of the study via email, and were invited to participate and share the survey. Adverts were also placed on the websites of Research Autism and Autism West Midlands. Participants who completed the whole survey were entered into a prize draw to win a £50 voucher.

A total of 111 individuals completed the survey, although 9 of these participants did not complete all of the demographic questions. 54 participants reported that they had a diagnosis of an autism spectrum condition (49%) and 73 participants reported a diagnosis of Asperger’s Syndrome (66%) - with overlap due to some participants selecting both options—which could reflect the current categorisation of Asperger’s Syndrome and autism under the umbrella category of “autism spectrum disorder” in the DSM-5 (APA 2013). Two participants reported a diagnosis of pervasive developmental disorder not otherwise specified. Further, 11 participants reported that they did not currently have a formal diagnosis of autism. These participants were not included in the regression analyses, since we controlled for age of diagnosis. However, we decided to include these individuals in other analyses since removing their responses did not alter any of the other results. Further, 84% self-reported additional mental health diagnoses or developmental conditions. This included depression (n = 57), anxiety (n = 62), social anxiety (n = 35), attention deficit hyperactivity disorder (n = 18), obsessive compulsive disorder (n = 18), post-traumatic stress disorder (n = 9), bipolar disorder (n = 7) and Tourette’s syndrome (n = 4).

Data collection took place from June 2016 to October 2016. The median time to complete the survey was 12 min. All participants gave full informed consent to participation and all responses were recorded anonymously. Ethical approval for this study was obtained through  Royal Holloway, University of London.

Participants were asked a number of demographic questions to establish the nature of the sample. The mean age of participants at the time of the survey was 36.4 (SD = 12.0), with a range from 18 to 72 years old. Mean age of diagnosis was 31.4 (SD = 14.0), ranging from 4 to 69 years old. Information on gender identity, sexual identity, employment, education and ethnicity is shown in Table 1. Demographic information demonstrated that the current sample mostly consisted of female, heterosexual, well-educated White British participants. Although women were not specifically targeted, the survey attracted substantially more autistic women than men. This could be due to women being more likely to complete surveys (Sax et al. 2003) or the topic being of particular interest to women.

**Table 1:** Demographic participant information on gender, sexual identity, employment, ethnicity and education (n = 104)

Variable	%*
Gender	
Male	27
Female	60
Transgender	1
Prefer not to say	1
Other**	12
Sexual identity	
Heterosexual	61
Gay/lesbian	7
Bisexual	10
Don’t know	6
Other***	17
Employment	
Full-time employment	18
Part-time employment	12
Self-employed	7
Unemployed	10
Unable to work	23
Retired	5
Student	16
Carer	8
Prefer not to say	2
Ethnicity	
White British	70
Other white background	18
Mixed ethnicity	4
Asian	1
Other	4
Prefer not to say	3
Highest level of education	
No qualifications	4
1 to 4 GCSEs or equivalent	9
5+ GCSEs or equivalent	7
Apprenticeship	1
2+ A-levels or equivalent	11
Undergraduate degree	31
Masters degree	24
Doctoral degree	3
Other qualifications	8
Prefer not to say	4

*Numbers may not add up to 100% due to rounding

**Participants reported a range of other gender identities such as a gender, non-binary or genderfluid

***Participants reported a range of other sexual identities such as asexual, pansexual and demi-sexual

### Materials and Procedure

Before the study commenced, the proposal for this research was reviewed by several autistic adults, to ensure that the research was in line with the priorities of the autism community—since all too often, autistic individuals are not involved in the research process itself (Pellicano et al. 2014). Feedback was positive and their advice was taken on board in ensuring question wording within the survey was clear and transparent.

Participants completed the survey online using the Qualtrics survey platform. Online survey methods were utilised in this research as this method is an efficient way to examine the suggested hypothesis. After giving consent, participants were asked for their preference of person-first (“person with autism”) or identity-first (“autistic person”) language, as there is debate around the use of these terms (Kenny et al. 2016). Results showed that 62% of our sample preferred identity-first language. Selection of preferred term meant that all of the survey questions reflected the individual’s preference where appropriate. Following this, participants first reported their diagnoses and age of autism diagnosis, followed by questions concerning experiences of acceptance, then the Depression, Anxiety and Stress Scale (DASS-21), and finally demographic questions.

#### Autism Acceptance Questions

To the best of our knowledge, there is no pre-existing measure designed to measure autistic individuals’ perceptions of autism acceptance. The current study aimed to quantify their perceptions of autism acceptance. First, participants were asked whether they felt that society (specified as the general public, made up of people who did not personally know them) generally accepted them, with “yes”, “no”, “sometimes” and “prefer not to say” as response options. These response options were used to obtain a categorical response for acceptance. Second, they were asked to rate the statement “over the past week, I have felt accepted by society as an autistic person/person with autism”, on a 5-point scale from “strongly agree” to “strongly disagree”. These response options were used to fit with the standardised 5-point scale used elsewhere in the survey (e.g. the DASS-21). Finally, to assess perceptions of autism acceptance from different sources, participants were also asked to report “how accepted by society do you feel as an autistic person”, “how accepted by your family and friends do you feel as an autistic person”, and “how much have you personally accepted yourself as an autistic person” on a scale from zero (“not at all”) to ten (“completely”). Validation of these items is discussed in the “Results” section.

Further, open textboxes were used to obtain qualitative responses, such that participants could “tell us more about [their] experiences of acceptance or non-acceptance”. Content analysis was subsequently conducted on these qualitative responses. Here, responses were first screened for common themes by two independent coders (JDM and VN) who familiarised themselves separately with the data. Categories were then agreed upon, discrepancies resolved and all responses were grouped into the categories by the two coders independently. Inter-rater reliability was calculated using the ReCal program (Freelon 2010) and is reported in the “Results” section.

#### Depression, Anxiety and Stress Scale (DASS-21)

The DASS-21 (Lovibond and Lovibond 1995) is a self-report scale used to measure depression, anxiety and stress. This measure consists of 21 items and is a short version of the full 42 item DASS. We used the short version as it is shown to have as good reliability and validity as the long version (Cronbach’s alpha 0.94, 0.87 and 0.91 for the depression, anxiety and stress subscales respectively, Antony et al. 1998; Ng et al. 2007), along with good construct validity (Henry and Crawford 2005). The DASS-21 has also previously been used with autistic adults (Maddox and White 2015). Participants were asked to rate 21 statements and to judge whether they could be applied to their life over the past week, on a scale from one to four (1 = did not apply to me at all; 2 = applied to me some of the time; 3 = applied to me a considerable degree; 4 = applied to me very much or most of the time). The 21 items could be divided into 7 items each for depression, anxiety and stress scales. A total score for each scale (depression, anxiety and stress) was computed and multiplied by two (maximum possible score for each scale = 42). Internal consistency in the current sample showed high consistency for the depression (Cronbach’s alpha = 0.919) and stress (Cronbach’s alpha = 0.842) scales, while the anxiety scale had acceptable consistency (Cronbach’s alpha = 0.790). These estimates are similar to previous research using the DASS with autistic adults (0.888, 0.861 and 0.805 for depression, stress and anxiety subscales respectively, Maddox and White 2015).

### Design

This cross-sectional study had a correlational design. The outcome variable was mental health (depression, anxiety or stress score on the DASS) and the main predictor variables of interest were perceptions of autism acceptance.

## Results

### Autism Acceptance

When asked whether they felt society, in general, accepted them as an autistic person, 7% of participants said “yes”, 43% said “no” and 48% said “sometimes” (2% preferred not to say). Participants were also asked to rate the statement “over the past week, I have felt accepted by society as an autistic person/person with autism”; 24.3% strongly agreed or agreed, 34% neither agreed nor disagreed, and 41.4% disagreed or strongly disagreed with this statement.

The validity of the three items examining autism acceptance from society, family and friends, and personal acceptance of autism diagnosis was assessed. As shown in Table 2, perceived acceptance from society and family and friends correlated significantly with ratings of acceptance from society over the past week. Personal acceptance did not correlate with this item or perceived societal acceptance, but did correlate with family and friend acceptance (r = .21, *p* = .027).

**Table 2:** Correlations between items measuring perceptions of autism acceptance

	Societal acceptance		Family and friend acceptance		Personal acceptance	
	*r*	*p*	*r*	*p*	*r*	*p*
Family and friend acceptance	0.485	< 0.001				
Personal acceptance	0.106	0.272	0.210	0.027		
Acceptance from society over past week*	− 0.721	< 0.001	− 0.440	< 0.001	− 0.102	0.288

*This item was scored according to how much the participant agreed that they had felt accepted by society as an autistic person over the past week on a 5-point scale from “strongly agree” (1) to “strongly disagree” (5). All other items were rated on a scale from “0” (not at all) to “10” (completely)

The three items assessing autism acceptance from the three sources were then assessed using principal component analysis (PCA) to test whether the items were measuring one overall construct of perceived acceptance, or if they were measuring acceptance from three separate sources. The KMO statistic was acceptable (0.528), individual item KMO values were above 0.74, and Bartlett’s test of sphericity was significant (χ^2^ (3) = 33.66, *p* < .001), indicating that PCA was appropriate with the current sample size (Field 2013). The analysis extracted one component (eigenvalue 1.57) explaining 52.46% of total variance. The factor loadings of the three items onto this component indicated that perceived acceptance from society (factor loading = 0.801) and family and friends (0.848) loaded significantly onto one component. However, personal acceptance did not load significantly onto the component (factor loading = 0.462; according to Stevens’ (2002, as cited in Field 2013) recommendation that only loadings greater than 0.512 for a sample size of 100 are statistically significant). Further, when testing for reliability using Cronbach’s alpha, removal of the personal acceptance item increased Cronbach’s alpha from 0.51 to a more acceptable value of 0.64. Given these results, the two items considering societal acceptance and family and friend acceptance were combined into a measure of “external sources of acceptance”, and that personal autism acceptance was considered as a separate construct.

Mean ratings of perceived acceptance, on a scale from zero (“not at all”) to ten (“completely”) from external sources (society, family and friends) and personal acceptance are shown in Table 3, showing that personal autism acceptance was greater than perceived acceptance from external sources, t(110) = 7.93, *p* < .001, 2-tailed, *d* = 0.76.

**Table 3:** Ratings of perceived autism acceptance from external sources (society, family and friends) and personal acceptance of own autism

	Mean (SD)
External sources	4.86 (2.23)
Societal acceptance	3.92 (2.32)
Family and friend acceptance	5.82 (2.84)
Personal acceptance	7.36 (2.91)

### Depression, Anxiety and Stress Scale

The mean for the DASS depression score was 17.8 (SD = 12.7), with scores ranging from 0 to 42. The mean for DASS anxiety score was 12.9, (SD = 9.14), ranging from 0 to 40. Finally, the mean DASS stress score was 22.3 (SD = 9.96), ranging from 0 to 42. These scores are similar to those reported in Maddox and White (2015) study with autistic adults (although they did not multiply the results by two), but are significantly higher than the means reported in the non-autistic population (for example, Henry and Crawford (2005) report the means from depression (5.66), anxiety (3.76) and stress (9.46) scores in 1794 non-autistic, non-clinical participants).

### Autism Acceptance and Mental Health

Considering the relationships between acceptance and mental health, Spearman’s correlational analyses showed that societal acceptance over the past week correlated significantly with DASS depression scores (ρ (107) = 0.288, *p* = .002) and DASS stress scores (ρ (109) = 0.281, *p* = .003); but there was no significant correlation with DASS anxiety scores (ρ (106) = 0.096, *p* = .32).

Further regression analyses were conducted to test whether acceptance from external sources or personal acceptance could predict mental health symptoms. Three separate hierarchical regressions with depression, anxiety and stress as the outcome variables were conducted. Blockwise entry was used to analyse the data; age, age of diagnosis, gender and the other DASS scales (since the scales were highly correlated with one another, Table 4) were entered into the first step for each model. In the second step, the two types of acceptance (external sources and personal) were entered. Several participants (n = 27) were not included in the final analyses as they had missing data for one or more of the variables. Correlations between the three DASS scales and predictor variables are shown in Table 4.

**Table 4:** Correlations between DASS scales and gender, age, age of diagnosis and acceptance items (n = 84)

	DASS depression		DASS anxiety		DASS stress	
	*r*	*p*	*r*	*p*	*r*	*p*
DASS anxiety	0.502	< **0.001**	–	–	–	–
DASS stress	0.557	< **0.001**	0.690	< **0.001**	–	–
Gender	− 0.039	0.364	− 0.069	0.267	− 0.024	0.414
Age	0.011	0.364	0.028	0.401	− 0.041	0.356
Age of diagnosis	0.034	0.379	0.142	0.099	0.061	0.290
External acceptance	− 0.497	< **0.001**	− 0.278	**0.005**	− 0.411	< **0.001**
Personal acceptance	− 0.411	< **0.001**	− 0.120	0.138	− 0.207	**0.029**

First, depression was considered as the outcome variable; the first step explained 34.6% of the variance in depression scores, and adding the acceptance variables as predictors in step two could explain 52.1% of the variance, a significant change (*p* < .001). The final model was also a significant fit to the data (F (7, 83) = 11.80, *p* < .001). This final model shows that age of diagnosis, anxiety, external acceptance and personal acceptance could significantly predict depression scores (Table 5).

**Table 5:** Hierarchical regression results with depression as the outcome variable

Predictor	B	SE B	β	*p*
Step one				
Age	0.234	0.229	0.225	0.310
Gender	0.095	1.10	0.008	0.931
Age of diagnosis	− 0.214	0.206	− 0.225	0.300
DASS anxiety	0.371	0.195	0.411	0.061
DASS stress	0.520	0.161	0.245	**0.002**
Step two				
Age	0.363	0.210	0.349	0.088
Gender	0.683	0.975	0.061	0.486
Age of diagnosis	− 0.368	0.182	− 0.387	**0.046**
DASS anxiety	0.431	0.170	0.285	**0.013**
DASS stress	0.277	0.150	0.219	0.068
External acceptance	− 1.65	0.554	− 0.284	**0.004**
Personal acceptance	− 1.43	0.408	− 0.307	**0.001**

*B* unstandardised beta coefficient, *SE B* standard error, *β* standardised beta coefficient

**Fig. 1:**
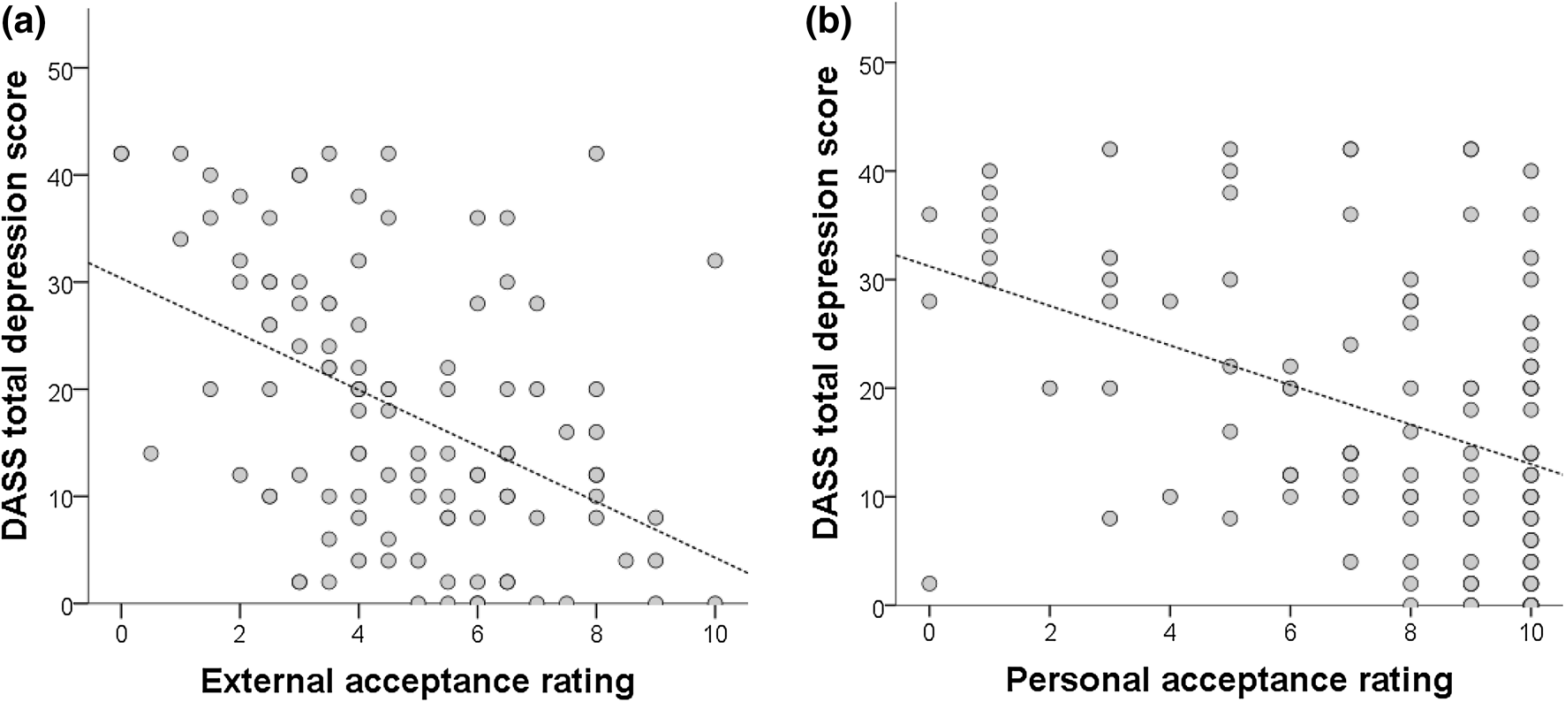
The relationship between depression scores and **a** autism acceptance from external sources and **b** personal autism acceptance

Figure 1 demonstrates the relationship between depression,  external acceptance and personal acceptance—indicating that there was a relationship between depression and autism acceptance such that greater levels of depression were associated with less perceived acceptance from external sources and less personal acceptance.

**Fig. 2:**
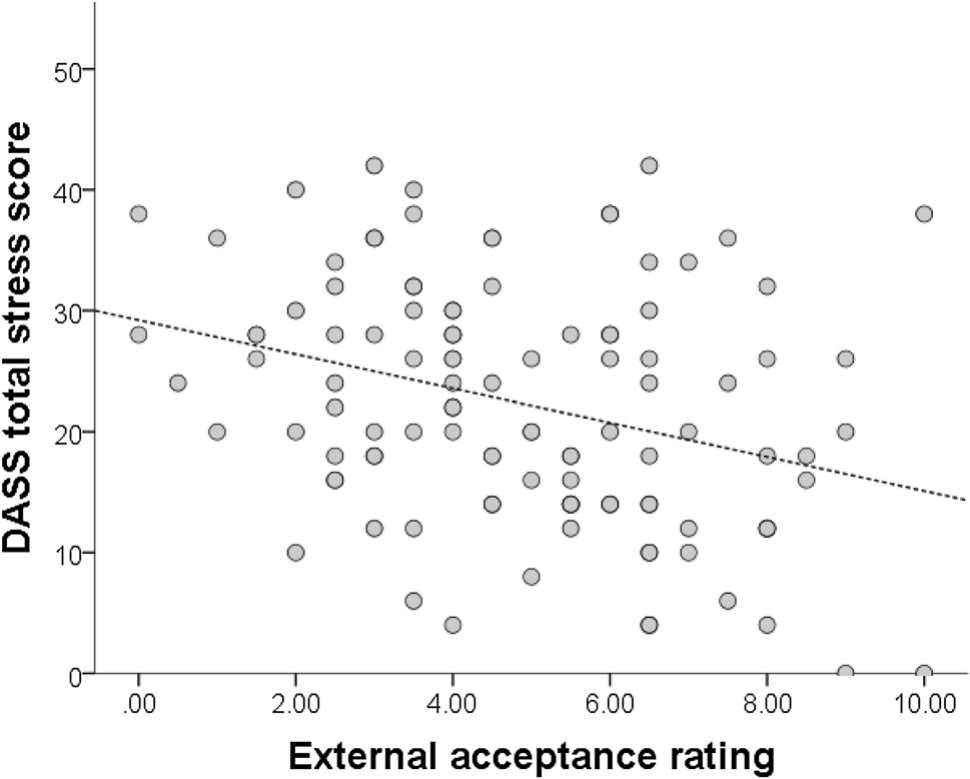
The relationship between stress scores and autism acceptance from external sources

Next, stress was entered as the outcome variable. The first step explained 54% of the variance in depression scores, and adding the acceptance variables in step two explained 57% of the variance, a non-significant change (*p* = .11). The final model was a significant fit to the data, F (7, 83) = 14.32, *p* < .001. This final model shows that anxiety and external acceptance significantly predicted stress scores (Table 6). With increasing stress scores, participants perceived less autism acceptance from external sources (Fig. 2).

**Table 6:** Hierarchical regression results with stress as the outcome variable

Predictor	B	SE B	β	*p*
Step one				
Age	− 0.155	0.151	− 0.189	0.308
Gender	− 0.032	0.725	− 0.004	0.965
Age of diagnosis	0.109	0.136	0.144	0.428
DASS anxiety	0.634	0.111	0.531	< **0.001**
DASS depression	0.227	0.070	0.287	**0.002**
Step two				
Age	− 0.180	0.159	− 0.219	0.262
Gender	− 0.114	0.733	− 0.013	0.877
Age of diagnosis	0.081	0.140	0.108	0.564
DASS anxiety	0.631	0.111	0.528	< **0.001**
DASS depression	0.156	0.084	0.197	0.068
External acceptance	− 0.908	0.427	− 0.198	**0.037**
Personal acceptance	0.053	0.329	0.015	0.871

*B* unstandardised beta coefficient, *SE B* standard error, *β* standardised beta coefficient

Finally, anxiety was tested as the outcome variable; here the first step explained 52% of the variance in depression scores, and adding the acceptance variables in step two explained 54% of the variance, a non-significant change (*p* = .21). The final model was a significant fit to the data, F (7, 83) = 12.61, *p* < .001. This final model shows that age of diagnosis, depression and stress scores significantly predicted anxiety scores (Table 7).

**Table 7:** Hierarchical regression results with anxiety as the outcome variable

Predictor	B	SE B	β	*p*
Step one				
Age	− 0.169	0.129	− 0.246	0.196
Gender	− 0.417	0.621	− 0.056	0.504
Age of diagnosis	0.194	0.115	0.309	0.096
DASS depression	0.119	0.063	0.181	0.061
DASS stress	0.468	0.082	0.560	< **0.001**
Step two				
Age	− 0.214	0.137	− 0.311	0.121
Gender	− 0.565	0.632	− 0.076	0.374
Age of diagnosis	0.241	0.118	0.383	**0**.**045**
DASS depression	0.182	0.072	0.275	**0.013**
DASS stress	0.474	0.083	0.566	< **0.001**
External acceptance	0.385	0.378	0.101	0.311
Personal acceptance	0.377	0.282	0.123	0.185

*B* unstandardised beta coefficient, *SE B* standard error, *β* standardised beta coefficient

### Content Analysis of Qualitative Results

63 participants gave qualitative responses when asked to provide further information on their experiences of autism acceptance. Content analysis was used to examine and categorise these responses, with four broad themes agreed upon: positive acceptance experiences, negative acceptance experiences, consequences of acceptance/non-acceptance, and an “other” category. Within these themes, 21 sub-categories were agreed. The categories are shown below in Table 8. Inter-rater reliability was assessed using kappa coefficients, demonstrating that 12 categories had almost perfect agreement, 7 had substantial agreement and 2 had moderate agreement (following the guidelines for agreement by Landis and Koch (1977)).

**Table 8:** Categories and sub-categories from content analysis of qualitative responses

Positive acceptance experiences	Negative acceptance experiences	Consequences of acceptance/non-acceptance	Other
→ From society	→ From society	→ Mental health difficulties	→ Impact of late diagnosis
→ From the autism community	→ From the autism community	→ Physical health difficulties	→ Unsure about acceptance
→ From specific organisations	→ From specific organisations	→ Feeling different and/or isolated	
→ From family/friends	→ From family/friends	→ Bullying	
→ From self	→ From self	→ Masking	
	→ Challenges of being an autistic woman	→ Need to educate more about autism	
	→ Misunderstandings and misconceptions	→ Difficulties with social interactions	

The categories with the most responses were misunderstandings and misconceptions about autism, experiences of masking/camouflaging, negative acceptance experiences from society and from specific organisations, mental health difficulties, and difficulties with social interactions. Example quotes from each of these categories are shown in Table 9.

**Table 9:** Example quotes in most frequently reported categories, alongside n for these categories

Category	Sub-category	n	Example quote(s)
Negative acceptance experiences	Misunderstandings and misconceptions	29	“People don’t seem to understand that autism affects every single aspect of who I am as a person, and telling me there should be a cure is telling me I shouldn’t exist... I can’t feel accepted by society until society understands that autistic people sometimes need support, and there’s nothing wrong with that, but there is something very wrong with wanting to change an autistic person into someone else entirely.” “Because my responses are slightly different from neurotypical people I am sometimes regarded as mentally ill when I am not. I find this very frustrating.”
	From society	13	“Generally I don’t think society accepts the traits that often go hand in hand with autism though and I therefore am also very pessimistic about the integration of people with autism into society.”
	From specific organisations (e.g. workplace, educational settings, etc.)	11	“Since being diagnosed I have found that, other than specific autism support services, mention of autism is met blankly or dismissed, even by those who have remarked on my oddity. I have told my employers, and they acknowledge what I have told them but don’t really understand what it means to me.”
Consequences of acceptance/non-acceptance	Experiences of camouflaging/masking	27	“I do not exhibit symptoms much or am able to mask/hide them almost completely” “I have to invest a lot of energy into “passing” as neurotypical” “I feel that I have spent the majority of my life engaged in the search for acceptance and therefore I can fake neurotypical behaviour pretty well.” “I mask well so I am accepted but not as an autistic person.”
	Mental health	8	“[Masking] is incredibly exhausting and stressful and has ultimately led to mental and physical health problems.” “As the years pass I suffer increasing anxiety for lack of even casual acceptance by my species and, conversely, huge spikes of anxiety when someone actually does ‘see’ me. Invisibility has become my comfort zone as well as my prison.”
	Difficulties with social interactions	8	“After a lifetime of observing people, trying to work out why I am different and so isolated, it seems to me that my lack of comprehension of non-verbal communication limits my interaction with NTs looking for expected responses and results in them looking past me.”

A subset of participants spontaneously reported experiences of “masking” or “camouflaging” the fact that they were on the autism spectrum (n = 27). These qualitative reports indicated a potential relationship between experiences of camouflaging and mental health, with some participants reporting how camouflaging had a detrimental effect on their psychological wellbeing. Exploratory analyses were thus conducted to examine the hypothesis that camouflaging could have negative effects on mental health. A two (camouflaging: yes or no) × three (DASS scale: depression, anxiety or stress) mixed ANOVA was conducted on DASS scores, to test whether camouflaging related to symptoms of mental health. There was a main effect of DASS (F (2, 212) = 37.45, *p* < .001, η_p_2 =0.26), with planned contrasts using Bonferroni showing that overall more depression was reported than anxiety (*p* < .001), but significantly less depression than stress (*p* = .037), as well as more stress than anxiety (*p* < .001). There was no main effect of camouflaging (F (1, 106) = 2.46, *p* = .12, η_p_2 = 0.023). There was a significant interaction between camouflaging and the DASS (F (2, 212) = 4.55, *p* = .012, η_p_2 = 0.041). Planned contrasts using t tests showed only significant differences in depression between those who reported camouflaging and those who did not (t (107) = − 0.256, *p* = .012, *g* = 0.56), with those who spontaneously reported camouflaging also reporting higher depression. Further exploratory analyses showed that when asked how accepted over the past week they had felt, those who reported camouflaging were more likely to disagree that they had experienced acceptance (Likelihood ratio: X_2_(2) = 6.68, *p* = .035). Finally, there was no significant gender differences in spontaneous reports of camouflaging (Likelihood ratio X_2_(2) = 0.60, *p* = .740).

## Discussion

The current study aimed to test the relationship between autism acceptance and mental health (specifically, depression, anxiety and stress) in autistic adults. We hypothesised that autistic adults who experienced less acceptance would show a greater prevalence of depression, anxiety and stress symptoms. Findings showed that depression was predicted by autism acceptance from external sources (society, family and friends) and personal acceptance. Stress was predicted only by acceptance from external sources. There was no relationship between anxiety and autism acceptance.

The finding that depression was predicted by acceptance from external sources makes sense if the importance of supportive others is first considered. For example, Hurlbutt and Chalmers (2002) claim that families have  a large role in helping autistic individuals develop the skills needed to become successful adults in society. Further, for adults and adolescents with Asperger’s Syndrome, Tantam (2000) argued that the care and support of family members who accept an individual’s anxiety can protect against the development of depression. Lasgaard et al. (2010) also noted that perceived social support from family and peers was negatively correlated with loneliness in autistic adolescents. Feeling accepted by others as an autistic person could be a protective factor against depression.

Further, we also found that greater personal autism acceptance predicted lower depressive symptoms. Indeed, a recent study by Cooper et al. (2017) showed that identifying with an autistic identity was positively associated with self-esteem, mediating an association with depression and anxiety. Cooper et al. (2017) suggest that autistic identity could act as a protective factor against mental health difficulties, which the current findings would support if personal acceptance is considered to be related to identity. It could be argued that the relationship between depression and personal acceptance is mediated by self-esteem—with acceptance serving to boost self-esteem and thus protect against depression. The possible relationship with self-esteem may also explain why only depression was associated with personal acceptance, since feelings of self-worth are part of depression but not anxiety or stress (APA 2013). Further, a meta-analysis of whether self-esteem predicted depression and anxiety in a non-autistic sample found the directional effect of self-esteem on depression was stronger than the effect of depression on self-esteem (Sowislo and Orth 2013). Future research considering acceptance should endeavour to measure self-esteem and clarify its relationship with mental health and autism acceptance.

We also found that stress was predicted by external sources of autism acceptance. Arguably, it is stressful to not be accepted by others. In the social psychology literature, social support has been shown to protect against stress (Haslam et al. 2005). For example, less stress is seen in workplaces where others are more accepting and supportive of whom an individual wants to be (Lang and Lee 2005). However, autistic individuals may struggle to feel accepted by various individuals - with research suggesting that they experience less social support from others as well as perceiving more stress (Bishop-Fitzpatrick et al. 2017). More research is needed to understand how autistic individuals’ experiences of acceptance might contribute to levels of stress, along with how external support might serve as protective factor.

In this study, participants’ qualitative responses add an additional dimension and further insight into their experiences. These responses revealed many in the sample “camouflaged”—in other words, they acted as though non-autistic or “neurotypical”. Being able to pretend or act as neurotypical fits with the idea that autistic individuals are capable of reputation management, and supports evidence that autistic individuals can present themselves in a specific light (Begeer et al. 2008; Cage et al. 2013, 2016a, b; Scheeren et al. 2010, 2015). Importantly, the current results indicate that this effort may be detrimental to mental health, with those who reported camouflaging also reporting higher symptoms of depression and fewer experiences of acceptance in the past week. Additionally, recent research has attempted to explain gender differences in the diagnosis of autism as females are suggested to be better at camouflaging than males (Bargiela et al. 2016; Dean et al. 2016; Lai et al. 2017; Tierney et al. 2016). However, the current study did not find any gender difference in spontaneous reports of camouflaging, with men just as likely to report camouflaging as women. More research is needed to further understand camouflaging and its relationship to mental health in autism.

Interestingly, autism acceptance did not predict anxiety. It could be the case that other non-social factors play more of a role in anxiety in autism. For example, intolerance of uncertainty is an important concept within anxiety disorders regardless of autism (Carleton et al. 2012), where being unable to deal with the uncertain aggravates anxiety. In both autistic children and adults, intolerance of uncertainty has been shown to relate to anxiety (Boulter et al. 2014; Maisel et al. 2016). Another factor which is thought to play an important role in anxiety in autism is sensory sensitivities (Green and Ben-Sasson 2010). Interestingly, Neil, Olsson and Pellicano (2016) found that intolerance of uncertainty *and* anxiety were related to autistic children’s sensory sensitivities, suggesting that there could be a dynamic interplay between different variables and mental health outcomes. More research is needed to examine a wide range of social and non-social risk factors for mental health difficulties in autism.

It is also worth considering how Theory of Mind ability may impact on perceptions of autism acceptance. It is hypothesised that autistic individuals have a difficultly with understanding other’s perspectives (Baron-Cohen et al. 1985), which could affect whether the individual can accurately recognise how accepted they are by other people. However, research has shown that autistic individuals can theorise about other minds, and this ability is dependent on cognitive ability (Bowler 1992), task demands (Peterson et al. 2013) and whether automatic or conscious Theory of Mind is being tested (Senju et al. 2009). A recent study by Heasman and Gillespie (2017) also demonstrated that neurotypical family members tended to underestimate their autistic family member’s perspective-taking ability. Arguably, it is therefore important that autistic people’s ability to reflect upon their experiences of autism acceptance is not underestimated. Mixed methods, as used in the current study, are a key way of validating and supporting autistic individuals’ experiences.

The current study is not without its limitations. First, online survey methods rely on self-report which may be deemed unreliable. As a means of testing an initial hypothesis, though, we believe that online survey methods are effective in reaching a large sample, in a means that is accessible to many autistic people. Second, the participants in this study were predominantly female, which may limit the generalisability of the findings. Despite this, gender was not a significant predictor in any of the analyses. Further, the experiences of autistic women have been overlooked (Pellicano et al. 2014), thus the reports from the current study are arguably of great value in helping enhance understanding of the experiences of autistic women, even if those experiences are not that different to men. Third, as well as autism, a high proportion of participants reported additional diagnoses which could suggest that the survey attracted autistic adults who had already experienced or were currently experiencing mental health difficulties, and the results may not be applicable to those who experience lower incidences of mental health problems. However, research shows that comorbid mental health difficulties are highly prevalent in autism (e.g. Eaves and Ho 2008), thus the current findings are relevant to a large proportion of the autistic population. Finally, the current sample also mainly consisted of well-educated individuals who would not be representative of the whole autism spectrum. More research is clearly needed to include a wider variety of individuals on the autism spectrum. Based on the current study’s limitations, future research should aim to access a wide range of autistic people using both online and offline surveys. Given the paucity of research into experiences of autism acceptance and its relationship to mental health, further in-depth qualitative research would also be advantageous for enhancing our understanding of these experiences.

Nonetheless, we believe that this study offers novel insight into the importance of autism acceptance for autistic adults and their mental health. Future research should further examine how mental health difficulties in autistic individuals can be protected against by improving autism acceptance. Interventions designed to improve family and/or peer support should be tested, as well as those intended to boost personal acceptance or self-esteem. For example, peer mentoring has been examined in a Higher Education context and been shown to have the potential to improve autistic students’ self-esteem (Lucas and James 2017). We would particularly advocate for interventions designed alongside autistic people, with a focus on neurodiversity (Gillespie-Lynch et al. 2017). Wider societal acceptance should also be strived for to reduce the need for autistic adults to camouflage, and instead be accepted as they are. Overall, the current study demonstrated relationships between experiences of autism acceptance, depression and stress in a sample of autistic adults. There is still a long way to go in understanding and tackling the high prevalence of mental health difficulties in autism, but we believe that the social model approach is a useful and positive lens through which mental health outcomes could be improved.

## Footnotes

1 We use identity-first language or “person on the autism spectrum” to fit with the language preferences of the autism community in the UK (Kenny et al. 2016) and the participants in the current study.

## References

[CR1] American Psychiatric Association (2013). Diagnostic and statistical manual of mental disorders.

[CR2] Antony MM, Bieling PJ, Cox BJ, Enns MW, Swinson RP (1998). Psychometric properties of the 42-item and 21-item versions of the Depression Anxiety Stress Scales in clinical groups and a community sample. Psychological Assessment.

[CR3] Bargiela S, Steward R, Mandy W (2016). The experiences of late-diagnosed women with autism spectrum conditions: An investigation of the female autism phenotype. Journal of Autism and Developmental Disorders.

[CR4] Baron-Cohen S, Leslie AM, Frith U (1985). Does the autistic child have a “theory of mind”?. Cognition.

[CR5] Baumeister RF, Leary MR (1995). The need to belong: desire for interpersonal attachments as a fundamental human motivation. Psychological Bulletin.

[CR6] Begeer S, Banerjee R, Lunenburg P, Meerum Terwogt M, Stegge H, Rieffe C (2008). Brief report: Self-presentation of children with autism spectrum disorders. Journal of Autism and Developmental Disorders.

[CR7] Bishop-Fitzpatrick L, Mazefsky CA, Eack SM (2017). The combined impact of social support and perceived stress on quality of life in adults with autism spectrum disorder and without intellectual disability. Autism.

[CR65] Boulter, C., Freeston, M., South, M., & Rodgers, J. (2014). Intolerance of uncertainty as a framework for understanding anxiety in children and adolescents with autism spectrum disorders. *Journal of Autism and Developmental Disorders, 44*(6), 1391–1402. 10.1007/s10803-013-2001-x 24272526

[CR8] Bowler DM (1992). “Theory of mind” in Asperger’s syndrome. Child Psychology & Psychiatry.

[CR9] Cage E, Bird G, Pellicano E (2016). Reputation management in children on the autism spectrum. Journal of Autism and Developmental Disorders.

[CR10] Cage E, Bird G, Pellicano E (2016). “I am who I am”: Reputation concerns in adolescents with autism. Research in Autism Spectrum Disorders.

[CR11] Cage E, Pellicano E, Shah P, Bird G (2013). Reputation management: evidence for ability but reduced propensity in autism. Autism Research.

[CR12] Carleton RN, Mulvogue MK, Thibodeau MA, McCabe RE, Antony MM, Asmundson GJ (2012). Increasingly certain about uncertainty: Intolerance of uncertainty across anxiety and depression. Journal of Anxiety Disorders.

[CR13] Chamberlain JM, Haaga DA (2001). Unconditional self-acceptance and psychological health. Journal of Rational-Emotive and Cognitive-Behaviour Therapy.

[CR14] Cooper K, Smith L, Russell A (2017). Social identity, self esteem, and mental health in autism. European Journal of Social Psychology.

[CR15] Dean M, Harwood R, Kasari C (2016). The art of camouflage: Gender differences in the social behaviours of girls and boys with autism spectrum disorder. Autism.

[CR16] Dillenburger K, Jordan JA, McKerr L, Devine P, Keenan M (2013). Awareness and knowledge of autism and autism interventions: A general population survey. Research in Autism Spectrum Disorders.

[CR17] Dillenburger K, McKerr L, Jordan JA, Devine P, Keenan M (2015). Creating an inclusive society… How close are we in relation to autism spectrum disorder? A general population survey. Journal of Applied Research in Intellectual Disabilities.

[CR18] Eaves LC, Ho HH (2008). Young adult outcome of autism spectrum disorders. Journal of Autism and Developmental Disorders.

[CR19] Field A (2013). Discovering statistics using IBM SPSS statistics.

[CR20] Flett GL, Besser A, Davis RA, Hewitt PL (2003). Dimensions of perfectionism, unconditional self-acceptance, and depression. Journal of Rational-Emotive and Cognitive-Behaviour Therapy.

[CR21] Freelon D (2010). ReCal: Intercoder reliability calculation as a web service. International Journal of Internet Science.

[CR22] Gardiner E, Iarocci G (2014). Students with autism spectrum disorder in the university context: Peer acceptance predicts intention to volunteer. Journal of Autism and Developmental Disorders.

[CR23] Gillespie-Lynch K, Kapp SK, Brooks PJ, Pickens J, Schwartzman B (2017). Whose expertise is it? Evidence for autistic adults as critical autism experts. Frontiers in Psychology.

[CR24] Gillott A, Standen PJ (2007). Levels of anxiety and sources of stress in adults with autism. Journal of Intellectual Disabilities.

[CR25] Green SA, Ben-Sasson A (2010). Anxiety disorders and sensory over-responsivity in children with autism spectrum disorders: Is there a causal relationship?. Journal of Autism and Developmental Disorders.

[CR26] Griffith GM, Totsika V, Nash S, Hastings RP (2012). ‘I just don’t fit anywhere’: support experiences and future support needs of individuals with Asperger syndrome in middle adulthood. Autism.

[CR27] Haslam SA, O’Brien A, Jetten J, Vormedal K, Penna S (2005). Taking the strain: Social identity, social support, and the experience of stress. British Journal of Social Psychology.

[CR28] Heasman B, Gillespie A (2017). Perspective-taking is two-sided: Misunderstandings between people with Asperger’s syndrome and their family members. Autism.

[CR29] Henry JD, Crawford JR (2005). The short-form version of the Depression Anxiety Stress Scales (DASS-21): Construct validity and normative data in a large non-clinical sample. British Journal of Clinical Psychology.

[CR30] Hull L, Petrides KV, Allison C, Smith P, Baron-Cohen S, Lai MC, Mandy W (2017). “Putting on My Best Normal”: Social camouflaging in adults with autism spectrum conditions. Journal of Autism and Developmental Disorders.

[CR31] Humphrey N, Lewis S (2008). Make me normal’: The views and experiences of pupils on the autistic spectrum in mainstream secondary schools. Autism.

[CR32] Hurlbutt K, Chalmers L (2002). Adults with autism speak out: Perceptions of their life experiences. Focus on Autism and Other Developmental Disabilities.

[CR33] Jaarsma P, Welin S (2012). Autism as a natural human variation: Reflections on the claims of the neurodiversity movement. Health Care Analysis.

[CR34] Kapp SK, Gillespie-Lynch K, Sherman LE, Hutman T (2013). Deficit, difference, or both? Autism and neurodiversity. Developmental Psychology.

[CR35] Kelly A, Barnes-Holmes D (2013). Implicit attitudes towards children with autism versus normally developing children as predictors of professional burnout and psychopathology. Research in Developmental Disabilities.

[CR36] Kenny L, Hattersley C, Molins B, Buckley C, Povey C, Pellicano E (2016). Which terms should be used to describe autism? Perspectives from the UK autism community. Autism.

[CR37] Lai, M. C., Lombardo, M. V., Ruigrok, A. N., Chakrabarti, B., Auyeung, B., Szatmari, P., … Baron-Cohen, S. (2017). Quantifying and exploring camouflaging in men and women with autism. *Autism*. doi:10.1177/1362361316671012. 10.1177/1362361316671012 PMC5536256 27899710

[CR38] Landis JR, Koch GG (1977). The measurement of observer agreement for categorical data. Biometrics.

[CR39] Lang JC, Lee CH (2005). Identity accumulation, others’ acceptance, job-search self-efficacy, and stress. Journal of Organizational Behaviour.

[CR40] Lasgaard M, Nielsen A, Eriksen ME, Goossens L (2010). Loneliness and social support in adolescent boys with autism spectrum disorders. Journal of Autism and Developmental Disorders.

[CR41] Lovibond SH, Lovibond PF (1995). Manual for the depression anxiety stress scales.

[CR66] Lucas, R., & James, A. I. (2017). An evaluation of specialist mentoring for University students with autism spectrum disorders and mental health conditions. *Journal of Autism and Developmental Disorders*. doi:10.1007/s10803-017-3303-1. 10.1007/s10803-017-3303-1 28918532

[CR42] Maddox BB, White SW (2015). Comorbid social anxiety disorder in adults with autism spectrum disorder. Journal of Autism and Developmental Disorders.

[CR43] Maisel ME, Stephenson KG, South M, Rodgers J, Freeston MH, Gaigg SB (2016). Modelling the cognitive mechanisms linking autism symptoms and anxiety in adults. Journal of Abnormal Psychology.

[CR44] Mak WW, Poon CY, Pun LY, Cheung SF (2007). Meta-analysis of stigma and mental health. Social Science & Medicine.

[CR45] Mazurek MO (2014). Loneliness, friendship, and well-being in adults with autism spectrum disorders. Autism.

[CR46] Milton D, Sims T (2016). How is a sense of well-being and belonging constructed in the accounts of autistic adults?. Disability & Society.

[CR47] Neil L, Olsson NC, Pellicano E (2016). The relationship between intolerance of uncertainty, sensory sensitivities, and anxiety in autistic and typically developing children. Journal of Autism and Developmental Disorders.

[CR48] Nevill RE, White SW (2011). College students’ openness toward autism spectrum disorders: Improving peer acceptance. Journal of Autism and Developmental Disorders.

[CR49] Ng F, Trauer T, Dodd S, Callaly T, Campbell S, Berk M (2007). The validity of the 21-item version of the Depression Anxiety Stress Scales as a routine clinical outcome measure. Acta Neuropsychiatrica.

[CR50] Pellicano E, Dinsmore A, Charman T (2014). What should autism research focus upon? Community views and priorities from the United Kingdom. Autism.

[CR51] Peterson CC, Slaughter V, Peterson J, Premack D (2013). Children with autism can track others’ beliefs in a competitive game. Developmental Science.

[CR52] Robertson, S. M. (2009). Neurodiversity, quality of life, and autistic adults: Shifting research and professional focuses onto real-life challenges. *Disability Studies Quarterly, 30*(1).

[CR53] Sasson NJ, Faso DJ, Nugent J, Lovell S, Kennedy DP, Grossman RB (2017). Neurotypical peers are less willing to interact with those with autism based on thin slice judgments. Scientific Reports.

[CR54] Sax LJ, Gilmartin SK, Bryant AN (2003). Assessing response rates and nonresponse bias in web and paper surveys. Research in Higher Education.

[CR55] Scheeren AM, Banerjee R, Koot HM, Begeer S (2015). Self-presentation and the role of perspective taking and social motivation in autism spectrum disorder. Journal of Autism and Developmental Disorders.

[CR56] Scheeren AM, Begeer S, Banerjee R, Meerum Terwogt M, Koot HM (2010). Can you tell me something about yourself? Self-presentation in children and adolescents with high functioning autism spectrum disorder in hypothetical and real life situations. Autism.

[CR57] Senju A, Southgate V, White S, Frith U (2009). Mindblind eyes: An absence of spontaneous theory of mind in Asperger syndrome. Science.

[CR58] Shakespeare T (2006). The social model of disability. The Disability Studies Reader.

[CR59] Sowislo JF, Orth U (2013). Does low self-esteem predict depression and anxiety? A meta-analysis of longitudinal studies. Psychological Bulletin.

[CR60] Stewart ME, Barnard L, Pearson J, Hasan R, O’Brien G (2006). Presentation of depression in autism and Asperger syndrome: A review. Autism.

[CR61] Tantam D (2000). Psychological disorder in adolescents and adults with Asperger syndrome. Autism.

[CR62] Tierney S, Burns J, Kilbey E (2016). Looking behind the mask: social coping strategies of girls on the autistic spectrum. Research in Autism Spectrum Disorders.

[CR63] Tipton LA, Blacher J (2014). Brief report: Autism awareness: Views from a campus community. Journal of Autism and Developmental Disorders.

[CR64] Weiss JA, Cappadocia MC, MacMullin JA, Viecili M, Lunsky Y (2012). The impact of child problem behaviours of children with ASD on parent mental health: The mediating role of acceptance and empowerment. Autism.

